# The effects of beliefs, knowledge, and attitude on herbal medicine use during the COVID-19 pandemic: A cross-sectional survey in Indonesia

**DOI:** 10.12688/f1000research.116496.3

**Published:** 2022-11-17

**Authors:** Heri Kristianto, Bayu Anggileo Pramesona, Yafi Sabila Rosyad, Lili Andriani, Tri Antika Rizki Kusuma Putri, Yohanes Andy Rias

**Affiliations:** 1 Department of Nursing, Faculty of Health Science, Universitas Brawijaya, Malang, Jawa Timur, 65145, Indonesia; 2 Department of Public Health, Faculty of Medicine, Universitas Lampung, Bandar Lampung, Lampung, 35145, Indonesia; 3 Department of Nursing, Faculty of Health and Medicine, Sekolah Tinggi Ilmu Kesehatan Yogyakarta, Yogyakarta, Yogyakarta, 55281, Indonesia; 4 Department of Pharmacy, Sekolah Tinggi Ilmu Kesehatan Harapan Ibu Jambi, Jambi, Jambi, 36122, Indonesia; 5 Department of Nursing, Faculty of Health and Medicine, Sekolah Tinggi Keperawatan PPNI Jabar, Bandung, Jawa Barat, 40173, Indonesia; 6 Department of Nursing, Faculty of Health, Institut Ilmu Kesehatan Bhakti Wiyata Kediri, Kediri, Jawa Timur, 64114, Indonesia; 7 Postdoctoral, Graduate School, Chulalongkorn University, Bangkok, Bangkok, 10330, Thailand

**Keywords:** Herbal medicine, holistic health belief, Indonesia, knowledge, magical health belief, pro-CAM attitude

## Abstract

**Background:** Herbal medicines are gaining a greater degree of popularity as complementary and alternative medicines during the COVID-19 pandemic. Nonetheless, there is a lack of data concerning the rationale for and factors influencing their use.

**Methods:** A cross-sectional community-based online study involving 1,621 participants was conducted to explore the effects of magical health beliefs, holistic health beliefs, knowledge, and pro- complementary alternative medicine (CAM) attitudes on herbal medicine use in the Indonesian population.

**Results:** Logistic regression findings showed that knowledge about herbal medicines was independently and positively associated with herbal medicine use to a greater extent than herbal medicine non-use (adjusted odds ratio; AOR = 1.20; 95% confidence interval; CI = 1.16 to 1.24). The participants who used herbal medicines had a greater magical health belief score than herbal medicine non-users, with AOR = 1.03 and 95% CI = 1.00 to 1.06. Moreover, holistic health beliefs and pro-CAM attitudes were also found to be independently associated with herbal medicine use.

**Conclusion:** Magical health beliefs, holistic health belief, knowledge, and attitudes are key factor in determining the herbal medicine use. Our findings offer crucial implications for health policymakers to encourage the use of herbal medicine during the COVID-19 pandemic.

## Introduction

The Coronavirus disease 2019 (COVID-19) pandemic has caused 4,621,205 deaths worldwide as of 12 September 2021 ( World Health Organization, 2021) and created widespread anguish, anxiety, and depression among the population ( Bueno-Notivol *et al.,* 2021; Nikčević *et al.,* 2021; Yıldırım *et al.,* 2021). This disease has spread throughout Indonesia, too, on 12 September 2021, with around 4,167,511 individuals afflicted and 138,889 deaths reported ( World Health Organization, 2021). Along with a sense of uncertainty and widespread false information ( Apuke & Omar, 2021; Chou *et al.,* 2021), this disease has prompted the population to seek and adopt remedies that promise to be effective at preventing contagion or death and at increasing immunity ( Gonzalez *et al.,* 2021; Moscatelli *et al.,* 2021), eventually leading to the use of herbal medicines ( Alyami *et al.,* 2020; Shahrajabian *et al.,* 2020).

Consumption of herbal medicines containing specific active substances with antibacterial or antiviral, anti-inflammatory, and immunomodulatory properties is a recent trend in the community ( Lee *et al.,* 2019; Nugraha *et al.,* 2020). These herbal medicines are believed to have the ability to modify the immune response and thus be effective at preventing COVID-19 ( Lee *et al.,* 2019; Nugraha *et al.,* 2020). Herbal medicines remain valuable sources for development of new medication, and their low toxicity makes them attractive prophylactic candidates for preventing COVID-19 ( Huang *et al.,* 2020). In Indonesia, herbal medicine consumption for the prevention of illnesses has grown and become more widespread ( Rahayu *et al.,* 2020). A national survey revealed an increase of 30.4% to 43.3% in household use of traditional healthcare products from 2013 to 2018 ( Kementerian Kesehatan Republik Indonesia, 2018). A few studies have assessed the prevalence and factors related to herbal medicine use in Indonesia ( Pengpid & Peltzer, 2018; Rahayu *et al.,* 2020). However, it is arduous to provide precise data on the use of herbal medicines because most recent studies targeted specific groups among the population in specific geographic distributions, thus their results cannot be generalized. Moreover, these reports did not specifically examine herbal medicine use for preventing COVID-19. In light of the current global health crisis, investigating the determinants of the use of herbal medicines is viable for prevention among the community during the COVID-19 pandemic.

Knowledge is a basic component of health practice modifications that measure public understanding of prevention efforts, particularly during a pandemic ( Abdulkareem *et al.,* 2020; Muslih *et al.,* 2021). Knowledge may be useful for discovering elements that contribute to the population developing a good attitude toward COVID-19 prevention and implementing healthy practices ( Alyami *et al.,* 2020). In the Ethiopian context, the population was familiar with the practice of using herbal medicines but faced challenges related to limited knowledge of herbal medicines ( Aina *et al.,* 2020). In Indonesia, with the spike in COVID-19 cases that is currently occurring in 2020, people are turning to herbal medicines to increase their immunity. However, the public health department warns that unproven treatment methods can lead to a false sense of security ( Hartanti *et al.,* 2020).

Herbal medicine use itself is most commonly driven by two reported reasons. First, herbal medicines are affordable ( Rahayu *et al.,* 2020; Sumarni *et al.,* 2021). Second, uniquely in Indonesia, they resonate more closely with patients’ beliefs, relieve concerns about the adverse effects of pharmaceutical medicines, and satisfy the desire for individualized health care ( Rahayu *et al.,* 2020; Sumarni *et al.,* 2021). Magical and holistic health beliefs are related to herbal medicine use ( Aarnio & Lindeman, 2004; Bryden *et al.,* 2018). The former increases concurrently with intuitive thinking, represented by affective information processing and the guidance of approach-avoidance behavior ( Aarnio & Lindeman, 2004). They are related to approach-avoidance behavior rather than making true-false distinctions about food and nutrition ( Aarnio & Lindeman, 2004; Bryden *et al.,* 2018). An instance of a magical health belief is the notion that consuming red beverages increases blood hemoglobin levels ( Bryden *et al.,* 2018). In contrast, the latter adheres to the philosophy that the entire individual must be regarded in maintaining health, including the mind, body, and spirit ( Bryden *et al.,* 2018). Unfortunately, both magical and holistic health beliefs have yet to be employed in an attempt to explain use of herbal medicines, particularly during the COVID-19 pandemic in Indonesia. Thus, a study of magical and holistic health beliefs in Indonesia should immediately be conducted.

Importantly, as existing literature suggests, attitudes toward complementary alternative medicine (CAM) use have been extensively studied in an attempt to comprehend preferences ( Berna *et al.,* 2019; Islahudin *et al.,* 2017). According to a previous study, around 41.1% of respondents with chronic illnesses preferred herbal medicines until these herbal medicines turned ineffective ( Berna *et al.,* 2019), which might represent widespread positive pro-CAM attitude. However, additional research is necessary to elucidate the underlying factors that contribute to stronger pro-CAM attitude. Considering that Indonesia is currently suffering from COVID-19 transmission and remains in a continuing battle against this pandemic ( Muslih *et al.,* 2021; Rias *et al.,* 2020), the association between knowledge related to herbal medicine use and pro-CAM attitude toward COVID-19 prevention needs to be assessed.

It is essential that accurate data be accessible to represent community opinions regarding the use of herbal medicine for health care. Therefore, the relationships between magical health beliefs, holistic health beliefs, knowledge, and attitudes toward CAMs on herbal medicine use during the COVID-19 pandemic should be ascertained. It is critical that health professionals, including the nursing community, be aware of the determinants of herbal medicine use, so that this information can be used in planning healthcare services. Therefore, the current study was conducted to investigate the prevalence of herbal medicine use and its determinant factors, such as magical health beliefs, holistic health beliefs, knowledge about herbal medicines, and pro-CAM attitude, in the case of the Indonesian population.

## Methods

### Study design and sample

**Figure 1.**
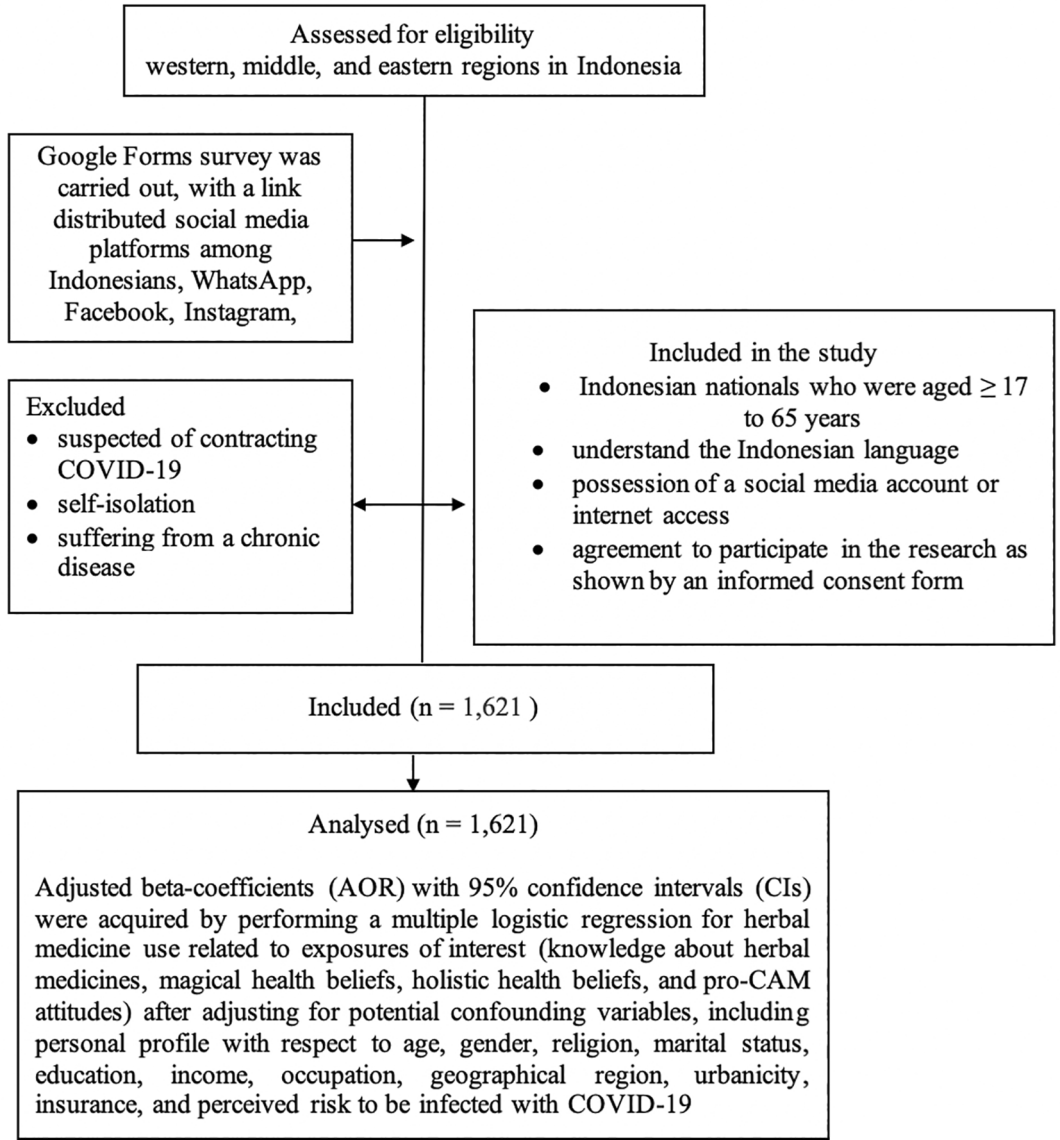
Methodology summarized of this study.

Primary data was collected as part of this cross-sectional study, with a community-based survey of a representative sample from the western, middle, and eastern regions of Indonesia ( Pusat Statistics Indonesia, 2019). Data collection was carried out from July 14 to September 12, 2021. As suggested by previous studies ( Muslih *et al.,* 2021; Rias *et al.,* 2020), a web-based recruitment strategy was employed to conduct convenience sampling. An online Google Forms survey was carried out, with a link distributed via the largest and most accessible social media platforms among Indonesians, WhatsApp, Facebook, Instagram, Telegram, and Twitter. This survey relied on the researchers' technological and personal networks. Social media influencers and community leaders also participated in and contributed to the survey ( Muslih *et al.,* 2021; Rias *et al.,* 2020). The inclusion criteria used were Indonesian nationals who were aged ≥ 17 to 65 years, able to understand the Indonesian language, in possession of a social media account or internet access, and in agreement to participate in the research as shown by an informed consent form. Meanwhile, the exclusion criteria were those who were suspected of contracting COVID-19, self-isolation, and suffering from a chronic disease as determined by Kementerian Kesehatan Republik Indonesia (2020). The total sample size was 1,621 ( Figure 1).

### Procedure

All questionnaires, including those on socio-demographic characteristics, knowledge about herbal medicines, magical health beliefs, holistic health beliefs, and complementary and alternative medicine attitude, were translated from English into Indonesian and validated by five experts to ensure the content validation, acceptability, and readability of the questions. Some modifications were made as per the feedback received to enable understanding of the questions. Two professionals from the nursing community independently translated the knowledge about herbal medicines, magical health beliefs, holistic health beliefs, and complementary and alternative medicine attitude from English to Bahasa Indonesia. These translations were then integrated into one and back-translated into English by another English/Bahasa translator professional and a native speaker from Indonesia without prior knowledge of the instrument. The content validity index of items was to confirm the integrity of a construct ( Wild *et al.,* 2005). The draft questionnaires were sent to a panel of five experts from the Universitas Andalas, Universitas Muhammadiyah Yogyakarta, and Universitas Muhammadiyah Surabaya, who have worked in fields relating to the subject of this study for over five years, including one specialist in infectious diseases, two experts on nursing community and two nurses with expertise in the complementary therapy field. Our survey was comprised of four major sections.

In the first section of the online survey, the respondents were given an explanation of the purpose of the survey and were asked for their consent to participate in the survey voluntarily. They were also given an explanation of their right to discontinue participation at any time and the survey’s privacy policy and details that their responses may be published. To proceed to the online survey, they were required to provide informed consent by checking the checkbox “agree” to confirm that they have read all information, including that the survey was set to be completed in a time period of 20 minutes. The second section comprised 11 questions related to socio-demographic characteristics. The third section consisted of one question that assessed the respondents’ herbal medicine use during the COVID-19 pandemic. Finally, the fourth section contained 27 questions across four questionnaires, including questionnaires on knowledge about herbal medicines, magical health beliefs, holistic health beliefs, and pro-CAM attitude. Upon completing the survey, the respondents would receive a thank you note, in which they were encouraged to persuade new Indonesian people from their contact list to take part in the survey. All responses were confidential and provided with informed consent. In ethical terms, this research was approved by the Survey and Behavioral Research Ethics Committee of *Institut Ilmu Kesehatan Strada Indonesia* (Reference number; 2271/KEPK/II/2021).

### Measurements

The demographic data collected included age, gender, religion, marital status, education, income, occupation, geographical region, urbanicity, insurance, and perceived risk of COVID-19 infection. Back-translation method was applied to the measuring instruments, i.e., questionnaires on knowledge about herbal medicines, magical health beliefs, holistic health beliefs, and pro-CAM attitudes, to translate the items from English into the Indonesian language and to ensure linguistic and conceptual equivalence in an item discriminant analysis with a *p* value of < 0.001. The questionnaires on knowledge of herbal medicines, magical health beliefs, holistic health beliefs, and pro-CAM attitude, in addition to the questionnaire on herbal medicine use, used the scales described below ( Rias, 2022b).

### Self-perceived knowledge about herbal medicines

The respondents’ knowledge about herbal medicines was assessed using the self-perceived knowledge questionnaire developed by Welz *et al.* (2019), containing 6 questions. This instrument is a 5-point Likert scale, ranging from 1 (very poor) to 5 (very good). A higher score indicates better self-perceived knowledge ( Welz *et al.,* 2019). The Cronbach’s (alpha) α of the instrument was 0.86, and, according to five nursing experts, the content validity value was 0.90 in our study.

### Magical health beliefs

Magical health beliefs were assessed with 10 items ( Lindeman *et al.,* 2000). A higher score indicates a higher level of magical health beliefs. The response options were “strongly disagree”, “somewhat disagree”, “somewhat agree”, and “strongly agree”, respectively scored 1, 2, 3, and 4. The Indonesian version of the instrument had a Cronbach’s α of 0.87. According to five nursing experts, the content validity value was 0.90 in current study.

### Holistic health belief

Holistic health beliefs were assessed using the holistic health beliefs model developed by Hyland *et al.* (2003). A higher score indicates a higher level of holistic health beliefs ( Hyland *et al.,* 2003). The response options were “strongly disagree”, “somewhat disagree”, “somewhat agree”, and “strongly agree”, respectively scored 1, 2, 3, and 4. In our study, the Indonesian version of the instrument had a Cronbach’s α of 0.90. According to five nursing experts, a content validity value of 0.95.

### Pro-CAM attitude

Assessment of pro-CAM attitude was concerned with the attitude of the participants toward the efficacy and desirability of CAMs ( Hyland *et al.,* 2003). The pro-CAM attitude scale included six questions: items 1, 2, 3, and 5 indicated negative questions, and items 4 and 6 indicated positive questions. In this study, the responses to the negative questions were reversed into positive questions. The four possible positive answers were “strongly disagree”, “somewhat disagree”, “somewhat agree”, and “strongly agree”, respectively scored 1, 2, 3, and 4. As a result, the total pro-CAM attitude score ranged from 6 to 24, with higher scores indicating a more pro-CAM attitude. The Indonesian version of the instrument had a Cronbach’s α of 0.78, and, according to five nursing experts, it had a content validity value of 0.95 in present study.

### Herbal medicine use

This was determined by questioning individual respondents about their personal use of herbal treatments to prevent or cure COVID-19-like symptoms. Herbal medicine included herbs or herbal products use was identified using the question: “During the COVID-19 pandemic, have you used any herbal medicine to prevent or cure COVID-19-like symptoms such as sore throat, flu, cough, fever, headache, or fatigue?” ( Rias, 2022b).

### Statistical analyses

Descriptive statistics were used to assess sociodemographic characteristics, knowledge of herbal medicines, magical health beliefs, holistic health beliefs, and pro-CAM attitude between groups. These variables were evaluated using χ ^2^ statistics or Fisher’s exact test, and the results are presented as percentages (%) and frequencies ( *n*). Continuous variables were evaluated using an independent *t*-test, and the results are presented as means and standard deviations (SD). The percentage of responses was established by counting the total number of participants per response for the total question. Multicollinearity was determined by calculating the variance inflation factor (VIF) (< 10) ( García *et al.,* 2015). This investigation yielded a maximum VIF of 2.56, indicating that multicollinearity effects were minimal. Adjusted beta-coefficients (AOR) with 95% confidence intervals (CIs) were acquired by performing a multiple logistic regression for herbal medicine use related to exposures of interest (knowledge about herbal medicines, magical health beliefs, holistic health beliefs, and pro-CAM attitudes) after adjusting for potential confounding variables, including personal profile with respect to age, gender, religion, marital status, education, income, occupation, geographical region, urbanicity, insurance, and perceived risk to be infected with COVID-19. SPSS, RRID:SCR_002865 version 25.0 (Chicago, IL) was used for all statistical analyses, and a *p*-value < 0.05 was considered statistically significant.

## Results

The overall sociodemographic characteristics of the participants are summarized in Table 1 ( Rias, 2022a). The sample included 1,621 participants, of whom 1,005 (62%) used herbal medicines and 616 (32%) did not use herbal medicines during the COVID-19 pandemic. χ ^2^ values showed that significant differences ( *p* < 0.05) were noted in age, religion, marital status, education, income, occupation, geographical region, insurance, and perceived risk to be infected with COVID-19 between herbal medicine users and herbal medicine non-users. However, no significant differences in gender or urbanicity were revealed between the groups.

**Table 1.** Relationships of distributions of demographic and determinant factors with use herbal medicine (HM) and HM non-user during COVID-19 pandemic (n=1621).

Characteristics	All participants n=1621, n (%)	HM non-user n=616, n (%)	HM user n=1005, n (%)	*P* value *
**Gender**				0.532
Male	507 (31.3)	187 (30.4)	320 (31.8)	
Female	1114 (68.7)	429 (69.6)	685 (68.2)	
**Age (years)**				<0.001
17–24	645 (39.8)	318 (51.6)	327 (32.5)	
25–39	634 (39.1)	223 (36.2)	411 (40.9)	
>40	342 (21.1)	75 (12.2)	267 (26.6)	
**Religion**				<0.001
Non-Islam	219 (13.5)	53 (8.6)	166 (16.5)	
Islam	1402 (86.5)	563 (91.4)	839 (83.5)	
**Marital status**				<0.001
Not married	833 (51.4)	388 (63.0)	445 (44.3)	
Married	788 (48.6)	228 (37.0)	560 (55.7)	
**Education**				<0.001
Elementary school	3 (0.2)	1 (0.2)	2 (0.2)	
Junior high school	36 (2.2)	1 (0.2)	35 (3.5)	
Senior high school	451 (27.8)	198 (32.1)	253 (25.2)	
Bachelor/Master/Doctoral	1131 (69.8)	416 (67.5)	715 (71.1)	
**Income (IDR)**				<0.001
<2.5 million	783 (48.3)	354 (57.5)	429 (42.7)	
2.5–5 million	561 (34.6)	183 (29.7)	378 (37.6)	
6–10 million	202 (12.5)	54 (8.8)	148 (14.7)	
>10 million	75 (4.6)	25 (4.1)	50 (5.0)	
**Occupation**				<0.001
Health professional	553 (34.1)	183 (29.7)	370 (36.8)	
Non‐health professional	450 (27.8)	126 (20.5)	324 (32.2)	
Unemployed	618 (38.1)	307 (49.8)	311 (30.9)	
**Geographical region**				
Western region	1378 (85.0)	580 (94.2)	798 (79.4)	<0.001
Central region	138 (8.5)	21 (3.4)	117 (11.6)	
Eastern region	105 (6.5)	15 (2.4)	90 (9.0)	
**Urbanicity**				0.260
Rural	614 (37.9)	244 (39.6)	370 (36.8)	
Urban	1007 (62.1)	372 (60.4)	635 (63.2)	
**Insurance**				<0.001
Yes	1289 (79.5)	452 (73.4)	837 (83.3)	
No	332 (20.5)	164 (26.6)	168 (16.7)	
**Perceived risk to be infected with COVID-19**				0.001
Not at all	90 (5.6)	47 (7.6)	43 (4.3)	
Low risk	371 (22.9)	161 (26.1)	210 (20.9)	
Moderate risk	420 (25.9)	156 (25.3)	264 (26.3)	
High risk	740 (45.7)	252 (40.9)	488 (48.6)	

Data are presented as the frequency and percentage, and *p* values were calculated using

^*^ Chi-Square test, with the *χ* ^2^-value in the bracket behind *p*-value. *p*<0.05 indicates statistical significance. IDR, Indonesian Rupiah; HM, Herbal Medicine.

We reveal the comparisons of knowledge about herbal medicines, magical health beliefs, holistic health beliefs, and pro-CAM attitude between herbal medicine users and herbal medicine non-users during the COVID-19 pandemic in Table 2. We observed that all items of self-perceived knowledge about herbal medicines differed significantly between groups. However, item-4 in holistic health belief and item-5 in pro-CAM attitude did not show any significant differences between groups. Interestingly, all items on the magical health beliefs questionnaire were significantly higher among herbal medicine users ( *p* < 0.01). The mean scores of knowledge about herbal medicines, magical health beliefs, holistic health beliefs, and pro-CAM attitude were significantly higher among the herbal medicine users’ group ( *p* < 0.01).

**Table 2.** Comparisons of citizen’s health knowledge, magical health belief, holistic health belief, and CAM attitudes with their herbal medicine (HM) use and HM non-user during COVID-19 pandemic (n = 1621).

Characteristics	HM non-user n=616, mean ± SD	HM user n=1005, mean ± SD	*P* value *
**Self-perceived knowledge about HM**	18.17 ± 4.25	20.63 ± 3.67	<0.001
Visual identification and differentiation of raw medicinal plants	3.05 ± 0.91	3.56 ± 0.73	<0.001
Medicinal effect and areas of application of raw medicinal herbs	3.31 ± 0.84	3.57 ± 0.78	<0.001
Medicinal effects and areas of application of processed HM products	3.06 ± 0.82	3.43 ± 0.76	<0.001
Potential unwanted side effects of raw or processed HM products	3.01 ± 0.78	3.42 ± 0.73	<0.001
Potential unwanted interaction effects with other HM products	2.96 ± 0.80	3.33 ± 0.74	<0.001
Safe dosage and safe use	2.95 ± 0.79	3.32 ± 0.73	<0.001
**Magical Health Belief**	30.70 ± 4.35	32.33 ± 4.18	<0.001
An imbalance between energy currents lies behind many illnesses	3.22 ± 0.65	3.36 ± 0.64	<0.001
Colors change the organism’s energy vibration in a direction that is beneficial to health	2.88 ± 0.67	3.10 ± 0.65	<0.001
Plants are living beings whose energy potentials can be transmitted to human beings	3.37 ± 0.66	3.50 ± 0.59	<0.001
By massaging diseased organs surrogate in the sole of the foot, the organ will be restored	3.06 ± 0.77	3.17 ± 0.77	0.004
An incorrect diet makes food rot in the body	2.88 ± 0.91	3.08 ± 0.89	<0.001
If we don’t somehow clean our bodies, unhealthy toxins remain in them	3.40 ± 0.72	3.53 ± 0.68	<0.001
It is good to detoxify one’s body every now and then with a fast	3.62 ± 0.59	3.75 ± 0.49	<0.001
An illness should be treated with a medicine that has properties similar to those of the illness	2.95 ± 0.74	3.18 ± 0.73	<0.001
Since our bodies are 70 percent water, we should be eating a diet that has an approximate water content of 70 percent	3.06 ± 0.77	3.17 ± 0.77	0.004
The statement that red drinks improve haemoglobin is probably valid.	2.27 ± 0.88	2.50 ± 0.94	<0.001
**Holistic Health Belief**	18.87 ± 1.82	19.17 ± 1.60	0.001
Positive thinking can help you fight off a minor illness	3.81 ± 0.50	3.88 ± 0.36	0.003
When people are stressed, it is important that they are careful about other aspects of their lifestyles as their body already has enough to cope with	3.61 ± 0.57	3.71 ± 0.53	0.001
The symptoms of an illness can be made worse by depression	3.82 ± 0.47	3.87 ± 0.41	0.029
If a person experiences a series of stressful life events, they are more likely to become ill	3.78 ± 0.49	3.82 ± 0.46	0.121
It is important to find a balance between work and relaxation in order to stay healthy	3.84 ± 0.43	3.89 ±0.36	0.015
**CAM Attitudes**	13.02 ± 2.36	14.21 ± 2.48	<0.001
Complementary medicine should be subject to more scientific testing before it can be accepted by conventional doctors (CAM 1)	1.28 ± 0.50	1.39 ± 0.67	<0.001
Complementary medicine can be dangerous in that it may prevent people getting proper treatment (CAM 2)	2.13 ± 0.83	2.41 ± 0.92	<0.001
Complementary medicine should only be used as a last resort when conventional medicine has nothing to offer (CAM 3)	2.28 ± 0.83	2.58 ± 0.93	<0.001
It is worthwhile trying complementary medicine before going to the doctor (CAM 4)	2.63 ± 0.79	2.84 ± 0.84	<0.001
Complementary medicine should only be used in minor ailments and not in the treatment of more serious illness (CAM 5)	2.02 ± 0.78	2.09 ± 0.93	0.100
Complementary medicine builds up the body’s own defences, so leading to a permanent cure (CAM 6)	2.69 ± 0.76	2.90 ± 0.85	<0.001

Data are presented as the mean ± SD, and p value were calculated using

^*^ independent sample *t-test.* A *p* value of <0.05 indicates statistical significance. CAM; Complementary and alternative medicine. Items CAM 1, 2, 3 and 5 was reverse score which indicated that a higher score on the CAM indicate more positive attitudes towards CAM.

Adjusted multiple logistic regression analyses showed that knowledge about herbal medicines, magical health beliefs, holistic health beliefs, and pro-CAM attitude were significantly associated with herbal medicine use (see Table 3). Knowledge about herbal medicines was found to be independently and positively associated with herbal medicine use to a greater degree than with non-use (AOR = 1.20; 95% CI = 1.16 to 1.24) after adjustment for confounding factors. Participants who used herbal medicines had a greater magical health belief score than non-users (AOR = 1.03 and 95% CI = 1.00 to 1.06). Moreover, holistic health beliefs and a pro-CAM attitude were found to be independently associated with herbal medicine use to a greater degree than with herbal medicine non-use during the COVID-19 pandemic after adjustment for confounding factors, including gender, age, religion, marital status, education, income, occupation, geographical region, urbanicity, insurance, and perceived risk of being infected COVID-19.

**Table 3.** Adjusted odds ratios (AOR) and confidence interval (%) for knowledge, magical health belief, holistic health belief, and CAM attitudes with their herbal medicine use and herbal medicine non-user during COVID-19 pandemic (n=1621).

Variables	Unadjusted OR (95% CI)	AOR (95%CI)
Knowledge about herbal medicine	1.18 (1.15–1.21) ^**^	1.20 (1.16–1.24) ^**^
Magical Health Belief	1.09 (1.07–1.12) ^**^	1.03 (1.00–1.06) ^*^
Holistic Health Belief	1.11 (1.05–1.18) ^*^	1.08 (1.01–1.60) ^*^
CAM Attitudes	1.23 (1.17–1.28) ^**^	1.24 (1.18–1.31) ^**^

Adjusted beta-coefficients (coef.) and 95% confidence intervals (CIs) were estimated using a multiple logistic regression after adjusting for gender, age, religion, marital status, education, income, occupation, geographical region, urbanicity, insurance, perceived risk to be infected COVID-19.

* A *p* value <0.05.

** *p* value <0.001. CAM; Complementary and alternative medicine.

## Discussion

In our study, we undertook a nationwide online survey of the Indonesian population to examine the prevalence of herbal medicine usage and to address its essential factors, including magical health beliefs, holistic health belief, knowledge about herbal medicines, and pro-CAM attitude, during the COVID-19 pandemic.

Our first notable finding addresses the herbal medicine use prevalence rate in the case of the Indonesian population during the COVID-19, which was found to be impressively high at 62% compared to those reported from other countries, including 49% in Vietnam ( Nguyen *et al.,* 2021), 30.8% in Turkey ( Karataş *et al.,* 2021), 22.1% in Saudi Arabia ( Alyami *et al.,* 2020), and 19.3% in Hong Kong ( Lam *et al.,* 2021). Why herbal medicine use prevalence estimate was higher in Indonesia than in other countries and why it was increasing were complex topics to discuss. However, one might conclude that cultural and societal contexts as well as individual attitudes and experiences might have a contribution to this high incidence of herbal medicine use ( Karataş *et al.,* 2021; Welz *et al.,* 2019). This could also be a result of the unique characteristics of the Indonesian healthcare system (e.g., health insurance and social security policies), traditional beliefs, and powerful herbal medicine advertising efforts ( Pengpid & Peltzer, 2018; Rahayu *et al.,* 2020). Throughout the COVID-19 pandemic, considerable emphasis and attention have been given to herbal medicines, with a rising body of evidence indicating that such approaches and preventive measures have been effective at fighting emerging infectious diseases.

Another finding further of this study demonstrates that knowledge about herbal medicines was positively correlated with herbal medicine use (AOR = 1.20; 95% CI = 1.16 to 1.24). Our finding is consistent with a study from Nigeria, where individuals who had experience using herbal medicines gained high scores of knowledge about herbal medicines ( Aina *et al.,* 2020). The beneficial impact of herbal medicine knowledge can enhance the positive attitude toward COVID-19 prevention ( Alyami *et al.,* 2020), as well as the awareness of side and interaction effects ( Welz *et al.*, 2019). It was assumed that these herbal medicines could possibly boost immunity and defend the body against COVID-19 infection ( Nugraha *et al.,* 2020; Panyod *et al.,* 2020). Herbal medicines were preferable to other complementary and alternative medical treatments due to their abundance and convenience of use ( Chaachouay *et al.,* 2021; Nguyen *et al.,* 2021; Panyod *et al.,* 2020). Additionally, these studies suggest that Indonesian people are more receptive and trusting of herbal therapies. Another factor in the use of herbal medications has been consumers' lack of knowledge about possible toxicity. It was indicated that most participants in our survey had a high level of knowledge of herbal therapies with total mean ± SD score was 20.63 ± 3.67. The negative impact of herbal medicine use was associated with lack of information regarding herb toxicity and drug-herb interactions. This is due to the fact that in such instances, the efficacy of such drugs is based solely on the therapeutic effects on the intended pathological condition, with very little information on side effects or toxicity ( Hartanti *et al.*, 2020). Since the first case of COVID-19 was found in Indonesia in March 2020, information about herbal remedies for COVID-19 protection has been extensively reported; as a result, Indonesians had adequate knowledge ( Badan Pengawas Obat dan Makanan Republik Indonesia, 2020; Nugraha *et al.,* 2020). In addition, with the historical and cultural herbal medicine use in Indonesia, it was presumed that a proper understanding of herbal medicines already exists ( Badan Pengawas Obat dan Makanan Republik Indonesia, 2020; Nugraha *et al.,* 2020). However, regulations and investigations of visual identification and differentiation of raw medicinal plants as well as safe dosages, safe uses, and side effects as aspects of knowledge of herbal medicines have become increasingly important ( Hartanti *et al.,* 2020). Therefore, strengthening knowledge about herbal medicines is critical for improving herbal medicine use in Indonesia during the COVID-19 pandemic.

Our research findings revealed that health beliefs, including holistic and magical health beliefs, were positively associated with the use of herbal medicine related to COVID-19 among the general population in Indonesia. In line with this, a recent research work, in Saudi Arabia, found that the majority of participants consumed herbal medicines during the pandemic era to enhance their immunity and minimize their risk of contracting COVID-19 infection ( Alyami *et al.,* 2020). It was also reported in another piece of previous research that there was a common belief that herbal medicines are both safer and of higher quality than prescription medications ( Barry, 2018; El Khoury *et al.,* 2016). In order to corroborate previously determined reasons, such as improved health and well-being with a reduction in unpleasant side effects associated with herbal medicines as well as improved holistic health beliefs, more research is required ( Rashrash *et al.,* 2017).

Magical health beliefs may aid in determining who is prone to hold such ideas and how magical thinking influences people's health behaviors and willingness or ability to deal with more abstract and intricate scientific knowledge regarding health ( Lindeman *et al.,* 2000). This study also suggests, there is a lack of evidence to connect health-related beliefs, such as magical or holistic health belief, to herbal medicine use. However, it is still reasonable to assume that these alternative medical beliefs may contribute to herbal medicine use. Lindeman and colleagues suggested that magical health beliefs, lacking empirical, logical, or scientific basis though they are, have an intuitive appeal due to assumptions about contagion, naturalness, and fundamental knowledge or ontological confusions ( Aarnio & Lindeman, 2004). Unlike magical health beliefs, holistic health beliefs are not demonstrably false or representative of cognitive biases or errors. For instance, the holistic health belief that it is critical to balance work and recreation to maintain good health is reasonable ( Nijp *et al.,* 2012). Many CAMs promote magical health beliefs and provide non-evidence-based food and health advice that follow magical truths ( Bryden *et al.,* 2018). A previous study has showed that magical and holistic health beliefs are associated with CAM attitudes ( Bryden *et al.,* 2018). Thus, the influence of generalized alternative health beliefs, such as magical food and health beliefs, and holistic health belief on herbal medicine use should be examined in Indonesia or another country with a high prevalence of herbal medicine use.

Unsurprisingly, Indonesian participants who scored high on pro-CAM attitude toward herbal medicine use were more likely to use herbal medicines (AOR = 1.24; CI 95% = 1.18–1.31). Similarly, a previous study in Vietnam presented a significant association between the attitudes toward herbal medicines and herbal medicine use during the COVID-19 pandemic ( Nguyen *et al.,* 2021). Attitude has been identified as the most important predictor of intention to use traditional Chinese medicines, including herbal medicines had significant correlation with attitude followed by previous actions (unstandardized path coefficient ( *β*) = 0.229, *p* < 0.001), subjective norms ( *β* = 0.190, *p* > 0.001), and perceived behavioral control ( *β* = 0.190, *p* > 0.001) ( Xia *et al.,* 2021). Prior research’s findings have significant implications for health policymakers interested in promoting the use of traditional medicines, which have helped to combat COVID-19 ( Xia *et al.,* 2021). People's positive attitudes toward herbal remedies were major factors in growing herbal medicine use ( Al Akeel *et al.,* 2018). In a separate study in Finland, individuals who used or showed favorable attitudes toward CAMs were found less likely to adhere to traditional therapies during the COVID-19 pandemic, but they demonstrated a high level of unwillingness to accept COVID-19 vaccine ( Soveri *et al.,* 2021). These findings emphasized the importance of developing trust-building communication strategies for the pros and cons of herbal medicine regulations ( Čavojová *et al.,* 2022; Soveri *et al.,* 2021). More generally, based on our findings, we suggest that increasing the positive value of pro-CAM attitude is a potentially effective strategy for improving herbal medicine use by developing proactive policies on safety, quality, and efficacy, as well as rational use.

There were several limitations to this investigation. First, the findings were built on self-reported data; consequently, respondents might have over- or under-reported their herbal medicine use. Second, as data collection was undertaken online, university-educated and younger individuals may have been overrepresented among the respondents. As this is an online survey, the study was done on a specific sample population. The population who did not have internet access was excluded. In future research, we suggest a face-to-face interview with an individual who does not have internet access. Another limitation is did not investigate the description of the type of herbal medicine (classical formulations or patent herbal drugs) and forms of herbal medicine (decoctions, pills, or crude powders). Moreover, the lack of participants from the central and eastern regions and persons who did not have insurance, all of whom may be recruited particularly in future studies, as this could affect the generalizability of the findings. However, we used multiple logistic regression analyses to account for a large number of potential confounding variables, thereby minimizing the influence of an unequal distribution.

The findings of this study provide nurses with information that will help them recognize herbal medicines as one of the most popular complementary and alternative medicines (CAMs) used by the general population to prevent the COVID-19 virus and to comprehend the cultural application of herbal medicines in the future. Additionally, determinant factors such as magical and holistic health beliefs, knowledge, and a pro-CAM attitude toward herbal medicine use may be suggested as primary factors for health care professionals such as nurses or community practice nurses collaborate with other health care such as pharmacists and medical doctors to explore alternative therapies in order to boost immunity and prevent infection of COVID-19. This study contributes to the understanding of the mechanisms of individual herbal medicine use. To begin with, the results indicated that respondents' with a pro-CAM attitude toward herbal medicine use had a considerable impact on their use. Additionally, both magical and holistic health beliefs were a major influence on herbal medicine use. Furthermore, knowledge about herbal medicines was associated with an increased likelihood of using herbal medicines. The present study's key findings have substantial practical implications for healthcare policymakers and professionals, particularly those tasked with developing programs to encourage and regulate herbal medicine use.

## Conclusion

The present research revealed that knowledge about herbal medicines, magical health beliefs, holistic health beliefs, and pro-CAM attitude were significantly associated with herbal medicine use during the COVID-19 pandemic. Specifically, we concluded that magical and holistic health beliefs were significant predictors of herbal medicine use. Knowledge about herbal medicines, including identified and potential side effects, interaction effect, safe dose, safe use, and raw materials, all played critical roles in predicting herbal medicine use. Finally, policymakers may use our findings to elevate knowledge and attitude as well as health beliefs to encourage the use of herbal medicines in a regulated manner to benefit public health.

## Data availability

### Underlying data

Figshare: The Effects of Beliefs, Knowledge, and Attitude on Herbal Medicine Use during the COVID-19 Pandemic, https://doi.org/10.6084/m9.figshare.19559662.v1 ( Rias, 2022a).

This project contains the following underlying data.

- Data_Herbal Use.xlsx

### Extended data

Figshare: Copies of all questionnaires used.pdf, https://doi.org/10.6084/m9.figshare.19618866.v1 ( Rias, 2022b).

This project contains the following extended data.

- Copies of all questionnaires used.pdf

Data are available under the terms of the Creative Commons Attribution 4.0 International license (CC-BY 4.0).

## Author contributions

**H. Kristianto** contributed to Conceptualization, Data Curation, Formal Analysis, Funding Acquisition, Investigation, Methodology, Visualization, Writing – Original Draft Preparation.

**B.A. Pramesona** contributed to Conceptualization, Investigation, Methodology, Visualization, Writing – Original Draft Preparation

**Y.S. Rosyad** contributed to Conceptualization, Data Curation, Investigation, Methodology, Writing – Original Draft Preparation

**L. Andriani** contributed to Conceptualization, Data Curation, Investigation, Methodology, Writing – Original Draft Preparation

**T.A.R.K.Putri** contributed to Conceptualization, Data Curation, Investigation, Methodology, Writing – Original Draft Preparation

**Y.A. Rias** contributed to Conceptualization, Data Curation, Formal Analysis, Investigation, Methodology, Project Administration, Supervision, Writing – Original Draft Preparation, Writing – Review & Editing
