# Supplementary material for: The effects of beliefs, knowledge, and attitude on herbal medicine use during the COVID-19 pandemic: A cross-sectional survey in Indonesia
Source: F1000Res. 2022 Nov 17;11:483. Originally published 2022 May 3. [Version 3] doi: 10.12688/f1000research.116496.3 (PMC9647142; doi:10.12688/f1000research.116496.3)
Supplement: Peer review history

# Peer review: Reviewer response for version 3

**DOI:** 10.5256/f1000research.141022.r156053

**Published:** 2022-11-18

**Authors:** Lalu Muhammad Irham

**Affiliations:** 1 Faculty of Pharmacy, University of Ahmad Dahlan, Yogyakarta, Indonesia

I have no further comments to make.

Is the work clearly and accurately presented and does it cite the current literature?

Yes

If applicable, is the statistical analysis and its interpretation appropriate?

I cannot comment. A qualified statistician is required.

Are all the source data underlying the results available to ensure full reproducibility?

Partly

Is the study design appropriate and is the work technically sound?

Yes

Are the conclusions drawn adequately supported by the results?

Yes

Are sufficient details of methods and analysis provided to allow replication by others?

Yes

Reviewer Expertise:

Genomic medicine, Pharmacogenomic

I confirm that I have read this submission and believe that I have an appropriate level of expertise to confirm that it is of an acceptable scientific standard.

# Peer review: Reviewer response for version 2

**DOI:** 10.5256/f1000research.136671.r152332

**Published:** 2022-11-09

**Authors:** Ansul Kumar, Zeya ul Haque

**Affiliations:** 1 CTVS Department, Rajendra Institute of Medical Sciences (RIMS), Ranchi, Jharkhand, India; 2 Rajendra Institute of Medical Sciences (RIMS), Ranchi, Jharkhand, India

This cross-sectional online survey-based study was conducted to explore various factors responsible for herbal medicine used in the Indonesian population during the COVID-19 pandemic. The roles of magical health beliefs, holistic health beliefs, knowledge of herbal medicine, and pro-attitudes toward herbal medicine were assessed. Logistic regression analysis reported that knowledge of herbal medicines, magical health beliefs, holistic health beliefs, and pro-attitudes showed a positive association with herbal medicine users than the herbal medicine non-user population.

 These findings may help health policymakers to include herbal drugs' availability in treatment centers. People should be encouraged to use herbal drugs with proven efficacy and safety, rather than the holistic belief approach. The herbal medications which were used mostly during the COVID-19 pandemic should be studied and standardized for their effect. Overall, the study design was good and holds enough important conclusions to be indexed.

 Below are my suggestions to be included if possible:

- The conclusion presented in the abstract should focus on the main findings, rather than suggesting the nurses to assess the role of magical health beliefs, holistic health beliefs, knowledge, and attitudes.
- The study was conducted among the general population in Indonesia. The assessment should be done on how much the individuals using herbal medicine suffered from COVID-19 infection during the pandemic period.
- The study lacks a description of the type of herbal medicine (classical formulations/ patent herbal drugs), and forms of herbal medicine (decoctions/pills/crude powders) used mostly by the sample population.
- As this is an online based survey, the study was done on a specific sample population. A major part of the population who did not have internet access has been excluded. Another study should be designed to include these people also.
- Any negative impact of using herbal medicine felt by the HM users should be analyzed and described in the study.

Is the work clearly and accurately presented and does it cite the current literature?

Yes

If applicable, is the statistical analysis and its interpretation appropriate?

Yes

Are all the source data underlying the results available to ensure full reproducibility?

Yes

Is the study design appropriate and is the work technically sound?

Partly

Are the conclusions drawn adequately supported by the results?

Yes

Are sufficient details of methods and analysis provided to allow replication by others?

Yes

Reviewer Expertise:

My areas of research are Herbal medicine, Traditional medicine,  COVID-19 disease, Cardiac disorders, Respiratory disease.

We confirm that we have read this submission and believe that we have an appropriate level of expertise to confirm that it is of an acceptable scientific standard.

# Response

**Published:** 2022-11-10

**Authors:** Yohanes Andy Rias

**[** **Version 2; peer review: 2** **]**

**Response to Reviewer Comments**

**Dear Reviewer**

 Thank you for considering our manuscript and for the valuable suggestions, also the opportunity to resubmit a revised manuscript, which helps us to improve the article. We carefully revised the manuscript in accordance with your comments. Our point-by-point responses to the comments are as follows. Thank you very much for your consideration.

**Point 1.** These findings may help health policymakers to include herbal drugs' availability in treatment centers. People should be encouraged to use herbal drugs with proven efficacy and safety, rather than the holistic belief approach. The herbal medications which were used mostly during the COVID-19 pandemic should be studied and standardized for their effect. Overall, the study design was good and holds enough important conclusions to be indexed.

**Response 1: **Thank you so much.

 Below are my suggestions to be included if possible:

**Point 2.** The conclusion presented in the abstract should focus on the main findings, rather than suggesting the nurses to assess the role of magical health beliefs, holistic health beliefs, knowledge, and attitudes.

**Response 2: **Thank you for your valuable comment. In order to make manuscripts to be better presented with precise, we re-organize the conclusion in the abstract based on the reviewer’s suggestion as follows; (please see conclusion in the abstract).

 “Magical health beliefs, holistic health belief, knowledge, and attitudes are key factor in determining the herbal medicine use. Our findings offer crucial implications for health policymakers to encourage the use of herbal medicine during the COVID-19 pandemic.”

**Point 3.** The study was conducted among the general population in Indonesia. The assessment should be done on how much the individuals using herbal medicine suffered from COVID-19 infection during the pandemic period.

**Response 3: **Thank you for your insightful feedback. Let us clarify this point. Please see study design and sample.

 “Meanwhile, the exclusion criteria were those who were suspected of contracting COVID-19, self-isolation, and suffering from a chronic disease as determined by Kementerian Kesehatan Republik Indonesia (2020)”.

**Point 4.**The study lacks a description of the type of herbal medicine (classical formulations/ patent herbal drugs), and forms of herbal medicine (decoctions/pills/crude powders) used mostly by the sample population.

**Response 4: **Thank you for your valuable suggestions. Our study did not evaluated the description of the type of herbal medicine (classical formulations/ patent herbal drugs) and forms of herbal medicine (decoctions/pills/crude powders). Consequently, we add this issue in the limitation section. Please see the limitation section.

 “Another limitation is did not investigate the description of the type of herbal medicine (classical formulations or patent herbal drugs) and forms of herbal medicine (decoctions, pills, or crude powders).”

**Point 5. **As this is an online based survey, the study was done on a specific sample population. A major part of the population who did not have internet access has been excluded. Another study should be designed to include these people also.

**Response 5: **Thank you for your valuable comment and suggestions. In this revised manuscript, we add the limitation study related the population who did not have internet access. Please see the limitation section.

 “As this is an online survey, the study was done on a specific sample population. The population who did not have internet access was excluded. In future research, we suggest a face-to-face interview with an individual who does not have internet access.”

**Point 6. **Any negative impact of using herbal medicine felt by the HM users should be analyzed and described in the study.

**Response 6: **Thank you for your insightful feedback. Let us clarify this point; the current study aims to investigate the prevalence of herbal medicine use and its determinant factors, such as magical health beliefs, holistic health beliefs, knowledge about herbal medicines, and pro-CAM attitude, in the case of the Indonesian population (between herbal medicine users and herbal medicine non-users) during COVID-19 pandemic. Consequently, we did not investigate any negative impact of using herbal medicine felt by the HM users. However, we investigated the knowledge about herbal medicines, including identified and potential side effects, interaction effect, safe dose, safe use, and raw materials, all played critical roles in predicting herbal medicine use. Please see in discussion section and conclusion.

# Peer review: Reviewer response for version 2

**DOI:** 10.5256/f1000research.136671.r146166

**Published:** 2022-08-08

**Authors:** Lalu Muhammad Irham

**Affiliations:** 1 Faculty of Pharmacy, University of Ahmad Dahlan, Yogyakarta, Indonesia

The manuscript improved significantly compared to the last version. The authors have addressed all questions and concerns.

Is the work clearly and accurately presented and does it cite the current literature?

Yes

If applicable, is the statistical analysis and its interpretation appropriate?

I cannot comment. A qualified statistician is required.

Are all the source data underlying the results available to ensure full reproducibility?

Partly

Is the study design appropriate and is the work technically sound?

Yes

Are the conclusions drawn adequately supported by the results?

Yes

Are sufficient details of methods and analysis provided to allow replication by others?

Yes

Reviewer Expertise:

Genomic medicine, Pharmacogenomic

I confirm that I have read this submission and believe that I have an appropriate level of expertise to confirm that it is of an acceptable scientific standard.

# Peer review: Reviewer response for version 1

**DOI:** 10.5256/f1000research.128328.r142022

**Published:** 2022-07-12

**Authors:** Lalu Muhammad Irham

**Affiliations:** 1 Faculty of Pharmacy, University of Ahmad Dahlan, Yogyakarta, Indonesia

**Review summary:**

 In this manuscript, the authors have tried to interlink the effect of beliefs, knowledge, and attitude on herbal medicine use during the COVID-19 pandemic. The manuscript is well written; however, the authors need to explicitly elaborate on a few points

**Comments & questions:**

1.  In the abstract part, the term “independently” has an ambiguous meaning. Please consider using more specific terms to avoid misinterpretation of meaning.
2. The conclusion of this study does not reflect the title e.g., the role of nurse health care in the herbal medicine used. In the title as well as in the abstract no explanation about the nurse's role but in the conclusion, the authors directly claim that findings alert nurses to assess the role of magical health, etc. please make sure about this.  
3.  I don’t find explicitly the finding of attitude toward herbal medicine used in the abstract part, please clarify this.
4.  In the introduction part. Please mention the full name of COVID-19 as the first mention of the abbreviation.
5. Please mention explicitly the accession date of the data of mortality such as the data of COVID-19 mortality “5.331.019”. as well as the data of mortality of Indonesian COVID-19 patients
  “The COVID-19 pandemic has caused **5,331,019** deaths worldwide (World Health Organization, 2021) and created widespread anguish, anxiety, and depression among the population (Bueno-Notivol et al., 2021; Nikcevic et al., 2021; Yıldırım et al., 2021). This disease has spread throughout Indonesia too, with around **4,260,148 individuals** afflicted and **143,986 deaths** reported (World Health Organization, 2021)”
6. Please cite this sentence “Consumption of herbal medicines containing specific active substances with antibacterial or antiviral, anti-inflammatory, and immunomodulatory properties is a recent trend in the community”.
7.  “These herbal medicines are believed to have the ability to modify the immune response and thus be effective at **preventing or treating** COVID-19 “(Lee *et al.,* 2019; Nugraha *et al.,* 2020). The author should carefully use terms of herbal medicine that can treat COVID-19. So far herbal medicine is just for prevention rather than treating the disease including COVID-19.
8.  I highly recommend to authors to use graphical with good visualization or graphical figure for elaborating the methodology then your readers will more easily understand the methodology that the authors used in the current study.
9. The respondents’ knowledge about herbal medicines was assessed using the self-perceived knowledge questionnaire developed by Welz *et al., *(2019), containing 6 questions.
  Reviewer concern here, does the author have permission to use this questionnaire? If you have permission please mention it
10. The authors should explain in more detail what type of herbal medicine that authors mean in this study? So, far as I know herbal medicine in Indonesia has several types.
11. “Another factor in the use of herbal medications has been consumers' lack of knowledge about possible toxicity “. Authors also should explain the negative impact of herbal medicine use. Authors can put this part in the discussion.
12.  Furthermore, knowledge about herbal medicines was associated with an increased likelihood of using herbal medicines.
  Reviewer’s concern here about the knowledge? The positive or negative impact of knowledge about herbal medicine? This should be clearly elaborated.
13. What is the role of nurse regarding the herbal medicine use?
14. Authors should also explain that the nurses should collaborate with other health care such as pharmacists, medical doctors, etc. to optimize the use of herbal medicine.

Is the work clearly and accurately presented and does it cite the current literature?

Yes

If applicable, is the statistical analysis and its interpretation appropriate?

I cannot comment. A qualified statistician is required.

Are all the source data underlying the results available to ensure full reproducibility?

Partly

Is the study design appropriate and is the work technically sound?

Yes

Are the conclusions drawn adequately supported by the results?

Yes

Are sufficient details of methods and analysis provided to allow replication by others?

Yes

Reviewer Expertise:

Genomic medicine, Pharmacogenomic

I confirm that I have read this submission and believe that I have an appropriate level of expertise to confirm that it is of an acceptable scientific standard, however I have significant reservations, as outlined above.

# Response

**Published:** 2022-07-25

**Authors:** Yohanes Andy Rias

**[Version 1; peer review: 1]**

**Response to Reviewer 1 Comments**

**Dear Reviewer #1**,

 Thank you for considering our manuscript and for the valuable suggestions, also the opportunity to resubmit a revised manuscript, which help us to improve the article. We carefully revised the manuscript in accordance with your comments. The revised sections of the manuscript are highlighted with yellow color. Our point-by-point responses to the comments are as follows. We very much hope the revised manuscript is accepted for publication in F1000 *. *Thank you very much for your consideration.

**Comments & questions:**

**Point 1.** In the abstract part, the term “independently” has an ambiguous meaning. Please consider using more specific terms to avoid misinterpretation of meaning.

**Response 1: **Thank you for your comments. We appreciated this reviewer’s comments. Let we clarify and revise this point. We used the term “Independently” When a variable is associated with an outcome after adjusting for multiple other potential prognostic factors (often after regression analysis), the association is independent.

**Point 2.** The conclusion of this study does not reflect the title e.g., the role of nurse health care in the herbal medicine used. In the title as well as in the abstract no explanation about the nurse's role but in the conclusion, the authors directly claim that findings alert nurses to assess the role of magical health, etc. please make sure about this. 

**Response 2: **Thank you for your valuable comments. We have already revised the conclusion section that refers to your suggestion (Please see conclusion section in abstract).

 “The use of herbal medicine during the COVID-19 pandemic was a common practice among Indonesian people. The roles of magical health beliefs, holistic health beliefs, knowledge, and attitudes toward the use of herbal medicine are highlighted by these findings”

**Point 3.** I don’t find explicitly the finding of attitude toward herbal medicine used in the abstract part, please clarify this.

**Response 3: **Thank you for your comments. We appreciated this reviewer’s comments. Let we clarify this point. We already mention the finding of attitude toward herbal medicine used in the abstract section (Please see last sentences in the results section).

 “Moreover………pro-CAM attitudes were also found to be independently associated with herbal medicine use”.

**Point 4.** In the introduction part. Please mention the full name of COVID-19 as the first mention of the abbreviation.

**Response 4: **Thank you for your comments and suggestion. We add the full name of COVID-19 as the first mention of the abbreviation in the abstract and introduction section (Please see in the abstract line 2 and introduction line 1).

**Point 5.** Please mention explicitly the accession date of the data of mortality such as the data of COVID-19 mortality “5.331.019”. as well as the data of mortality of Indonesian COVID-19 patients

 “The COVID-19 pandemic has caused 5,331,019 deaths worldwide (World Health Organization, 2021) and created widespread anguish, anxiety, and depression among the population (Bueno-Notivol et al., 2021; Nikcevic et al., 2021; Yıldırım et al., 2021). This disease has spread throughout Indonesia too, with around 4,260,148 individuals afflicted and 143,986 deaths reported (World Health Organization, 2021)”

**Response 5: **Thank you for your comments and suggestion. In this revised manuscript, we add the accession date and new number based on the date of the data on mortality in the world and Indonesia.

 “The Coronavirus disease 2019 (COVID-19) pandemic has caused 4,621,205 deaths worldwide as of 12 September 2021”.

 “This disease has spread throughout Indonesia, too, on 12 September 2021, with around 4,167,511 individuals afflicted and 138,889 deaths reported.”

**Point 6.** Please cite this sentence “Consumption of herbal medicines containing specific active substances with antibacterial or antiviral, anti-inflammatory, and immunomodulatory properties is a recent trend in the community”.

**Response 6: **Thank you for your comments and suggestion. We add the references based on the reviewer’s suggestion.

 “Consumption of herbal medicines containing specific active substances with antibacterial or antiviral, anti-inflammatory, and immunomodulatory properties is a recent trend in the community ( Lee *et al.,* 2019; Nugraha *et al.,* 2020)”

**Point 7.** “These herbal medicines are believed to have the ability to modify the immune response and thus be effective at preventing or treating COVID-19 “(Lee et al., 2019; Nugraha et al., 2020). The author should carefully use terms of herbal medicine that can treat COVID-19. So far herbal medicine is just for prevention rather than treating the disease including COVID-19.

**Response 7: **Thank you for your comments and suggestion. In the revised manuscript, we deleted “treating” based on the reviewer’s suggestion to avoid debate information.

**Point 8.** I highly recommend to authors to use graphical with good visualization or graphical figure for elaborating the methodology then your readers will more easily understand the methodology that the authors used in the current study.

**Response 8: **Thank you for your comments and suggestion. We add the figure of methodology.

**Point 9.** The respondents’ knowledge about herbal medicines was assessed using the self-perceived knowledge questionnaire developed by Welz et al., (2019), containing 6 questions. Reviewer concern here, does the author have permission to use this questionnaire? If you have permission please mention it

**Response 9: **Thank you for your comments. We already permission to use knowledge about herbal medicines questionnaire. Moreover, the questionnaire already publish and added in supplementary information.

 Welz AN, Emberger-Klein A, Menrad K: The importance of herbal medicine use in the German health-care system: prevalence, usage pattern, and influencing factors. *BMC Health Serv. Res.* 2019;19(1):911–952. 31823758 10.1186/s12913-019-4739-0

**Point 10.** The authors should explain in more detail what type of herbal medicine that authors mean in this study? So, far as I know herbal medicine in Indonesia has several types.

**Response 10: **Thank you for your comments. We add information the type of herbal medicine included herbs or herbal product in the herbal medicine use section based on the reviewer’s suggestion.

**Point 11.** “Another factor in the use of herbal medications has been consumers' lack of knowledge about possible toxicity “. Authors also should explain the negative impact of herbal medicine use. Authors can put this part in the discussion.

**Response 11: **Thank you for your suggestions. In this revised manuscript, we add the negative impact of herbal medicine use without depleting the effectiveness of herbal medicine in the discussion section based on the reviewer’s suggestion.

 “The negative impact of herbal medicine use was associated with lack of information regarding herb toxicity and drug-herb interactions. This is due to the fact that in such instances, the efficacy of such drugs is based solely on the therapeutic effects on the intended pathological condition, with very little information on side effects or toxicity.”

**Point 12.** Furthermore, knowledge about herbal medicines was associated with an increased likelihood of using herbal medicines. Reviewer’s concern here about the knowledge? The positive or negative impact of knowledge about herbal medicine? This should be clearly elaborated.

**Response 12: **Thank you for your suggestions. In this revised manuscript, we add the positive impact of herbal medicine use in the discussion section based on the reviewer’s suggestion.

 “The beneficial impact of herbal medicine knowledge can enhance the positive attitude toward COVID-19 prevention (Alyami *et al.,* 2020), as well as the awareness of side and interaction effects (Welz *et al.,* 2019). It was assumed that these herbal medicines could possibly boost immunity and defend the body against COVID-19 infection (Nugraha *et al.,* 2020; Panyod *et al.,* 2020)”

**Point 13.** What is the role of nurse regarding the herbal medicine use?

**Response 13: **Thank you for your suggestions. In this revised manuscript, we clarify the role of nurse regarding the herbal medicine use in the discussion section based on the reviewer’s suggestion.

 “The findings of this study provide nurses with information that will help them recognize herbal medicines as one of the most popular complementary and alternative medicines (CAMs) used by the general population to prevent the COVID-19 virus and to comprehend the cultural application of herbal medicines in the future.”

 “Additionally, determinant factors such as magical and holistic health beliefs, knowledge, and a pro-CAM attitude toward herbal medicine use may be suggested as primary factors for health care professionals such as nurses or community practice nurses collaborate with other health care such as pharmacists and medical doctors to explore alternative therapies in order to boost immunity and prevent infection of COVID-19.”

**Point 14.** Authors should also explain that the nurses should collaborate with other health care such as pharmacists, medical doctors, etc. to optimize the use of herbal medicine.

**Response 14: **Thank you for your suggestions. In this revised manuscript, we clarify add the new information related the nurses should collaborate with other health care in the discussion section based on the reviewer’s suggestion.

 “Additionally, determinant factors such as magical and holistic health beliefs, knowledge, and a pro-CAM attitude toward herbal medicine use may be suggested as primary factors for health care professionals such as nurses or community practice nurses collaborate with other health care such as pharmacists and medical doctors to explore alternative therapies in order to boost immunity and prevent infection of COVID-19.”
